# Survival analysis of pelvic lymphadenectomy alone versus combined pelvic and para-aortic lymphadenectomy in patients exhibiting endometrioid type endometrial cancer

**DOI:** 10.3892/ol.2014.2653

**Published:** 2014-10-31

**Authors:** TAYFUN TOPTAS, TAYUP SIMSEK

**Affiliations:** Department of Obstetrics and Gynaecology, Division of Gynaecologic Oncological Surgery, Akdeniz University Hospital, Antalya, Konyaaltı 07070, Turkey

**Keywords:** endometrial cancer, endometrioid type, lymphadenectomy, survival

## Abstract

The therapeutic benefit of lymphadenectomy in patients exhibiting endometrial cancer (EC) remains controversial. The aim of the present study was to determine whether the addition of para-aortic lymphadenectomy to pelvic lymphadenectomy (PLND) improves survival in patients with endometrioid type EC. A single tertiary-center, retrospective analysis was conducted in a total of 186 patients who were surgically treated with either PLND alone (n=97) or combined pelvic and para-aortic lymphadenectomy (PPaLND; n=89). Adjuvant treatments were assigned according to the Gynecologic Oncology Group (GOG) risk of recurrence analysis. The primary endpoint of the present study was progression-free survival (PFS). The median follow-up time was 38 months (95% confidence interval, 36.47–42.90) for all patients. No statistically significant differences were identified between the two groups in terms of overall survival (OS), PFS or time to progression (TTP). Kaplan-Meier estimates of three-year OS, PFS and TTP for patients with low or low-intermediate risk were as follows: PLND, 100, 98.7 and 98.7%, respectively; and PPaLND, all 100%. The estimated three-year OS, PFS and TTP for patients with high or high-intermediate risk were as follows: PLND, 92.3, 81.3 and 81.3%; and PPaLND, 90.7, 77.1 and 80.9%, respectively. No statistically significant differences were detected in the three-year OS, PFS and TTP between the lymphadenectomy groups, regardless of the GOG risk of recurrence (PLND, 98.4, 95.3 and 95.3%; and PPaLND, 94.9, 87.1 and 89.4%). Therefore, the combination treatment, PPaLND did not provide any survival advantage over pelvic lymphadenectomy alone.

## Introduction

The management of endometrial cancer (EC) has significantly changed over the past 25 years. In 1988, EC staging was developed from a clinical to a comprehensive surgical staging system (1). Twenty years later, the International Federation of Gynaecology and Obstetrics (FIGO) committee revised the staging criteria based upon survival similarities or disparities among particular substages (2). Surgical staging provides pathological and prognostic data, and identifies those patients that require adjuvant treatment. However, FIGO did not define the precise or optimal anatomical borders for the performance of lymphadenectomies, nor the adequate number of lymph nodes (LN) that require removal for the comprehensive completion of the procedure. Due to a lack of consistency among recommendations, the extent of lymphadenectomy for EC in current practice worldwide varies from the limited procedure of LN sampling alone to combined pelvic and para-aortic lymphadenectomy (PPaLND) up to the renal vessels.

An additional indicator, which may be adopted for surgical staging is the potential therapeutic effect of a lymphadenectomy. Previous studies have assessed the survival effect of a lymphadenectomy in EC patients, with the majority of retrospective analyses demonstrating a survival benefit, particularly in patients presenting with node-positive disease (3–5), although two large randomized controlled trials (RCTs) revealed no evidence of survival benefit in patients with presumed early-stage disease (6,7). However, all of the previous studies are heterogeneous with regard to the tumor histology.

In the present study, clinical data were evaluated to determine whether combining para-aortic lymphadenectomy with pelvic lymphadenectomy (PLND) improves survival in patients exhibiting endometrioid type EC. To the best of our knowledge, this is the first study that addresses the survival effect of PPaLND in a single type of tumor histology.

## Patients and methods

### Study design and patients

A total of 276 patients with EC who underwent surgery at Akdeniz University Hospital (Antalya, Turkey) between January 2005 and August 2012 were included in the current retrospective study. In this study, tumor grading was performed according to the World Health Organization grading system (8) and tumor staging was performed according to the FIGO 2009 criteria (2). Demographic, clinicopathological and survival data, as well as information regarding the age at surgery, date and type of surgical procedure, histological type, tumor size, tumor grade, depth of myometrial invasion, lympho-vascular space invasion (LVSI), number of LNs removed, LN involvement, disease stage, coexistence of primary synchronous malignancy, adjuvant treatment, disease recurrence or progression, survival status, and the date of last follow-up were extracted from the institutional database, surgery notes and patient charts following approval from the ethics committee of Akdeniz University Hospital. Written informed consent was obtained from all patients.

The inclusion criteria were as follows: i) Endometrioid tumor histological type and ii) a surgical procedure that was performed via laparoscopy or laparotomy, which included a total hysterectomy and bilateral salpingo-oophorectomy, as well as either systematic PLND or PPaLND. Patients exhibiting a non-endometrioid histology, carcinosarcoma or primary synchronous malignancy, or that had not undergone LN dissection or had no survival data were excluded. Selective LN sampling was not considered to be LN dissection.

### Procedures

Systematic PLND is performed in all EC patients as a routine procedure at Akdeniz University Hospital, regardless of any predefined surgical risk factors. The dissected lymphatic basin sites during PLND were the external iliac, obturatory, internal iliac and inferior common iliac regions. The upper dissection margin was 2–3 cm above the iliac bifurcation. The decision to perform a para-aortic dissection was at the discretion of the surgeons. Systematic PPaLND included PLND plus removal of all LNs from the superior common iliac, pre-sacral, para-caval, pre-caval, inter aorta-caval, pre-aortic and para-aortic areas up to the renal vessels. The type or extent of systematic lymphadenectomy (PLND vs. PPaLND) varied among the practitioners over the study period even in the same individuals. The superior margin of the PPaLND was taken as the inferior mesenteric artery for certain patients, due to technical complications, including adhesions resulting from previous abdominal surgery, the type of incision made (transverse vs. midline), anatomical variation or morbid obesity.

The patients who underwent surgery prior to 2009 were restaged according to the FIGO 2009 criteria (2). The postoperative risk of recurrence stratification was determined by the following standards determined by Gynecologic Oncology Group (GOG)-249 protocol (9): Low risk (LR; stage IA, grade 1, LVSI-negative); low-intermediate risk (LIR; stages IA, IB and II that do not meet the LR or high-intermediate risk criteria); high-intermediate risk (HIR; stages IA, IB and II, with the following risk factors: i) Grade 2 or grade 3; ii) LVSI-positive; iii) outer half myometrial invasion; (iv) patient aged ≥70 years with one other risk factor; (v) patient aged ≥50 years with two other risk factors; (vi) any age with all three risk factors); and high risk (HR; stages III and IV).

According to institutional procedure, the EC postoperative adjuvant treatment strategy was as follows: Observation for LR patients, high-dose rate (HDR) brachytherapy alone for LIR patients, external pelvic irradiation alone for HIR patients and chemotherapy alone for HR-stage IVB patients. These procedures were consistent between the two lymphadenectomy groups.

In the PPaLND group, the patients with HR-stage IIIA to IIIC_1_ disease were treated with pelvic irradiation with or without chemotherapy, and patients with HR-stage IIIC_2_ disease were treated with extended field irradiation of the pelvic and para-aortic regions with or without chemotherapy. In the PLND group, the patients with HR-stage III disease were offered a comparible treatment strategy to those with HR-stage IIIC_2_ disease in the PPaLND group. The decision to administer chemotherapy depended on the preference of the patient.

The dose of HDR brachytherapy used in LR patients was 2,100 cGy to a 5-mm depth in three fractions (700 cGy per fraction). A dose of 1,500 cGy was applied in three fractions for HR patients following external beam irradiation. Patients with HIR were treated with external pelvic irradiation at a dose of 4,500–5,040 cGy in 25–28 fractions. A dose of 4,500 cGy was administered in 25 fractions simultaneously with pelvic irradiation in patients who received extended field irradiation to the para-aortic region. In HR patients with stage III disease, adjuvant chemotherapy consisted of three to four cycles of carboplatin (area under the curve, 5) and paclitaxel (175 mg/m^2^) administered every three weeks. The same regimen was provided for six cycles in patients with stage IVB disease.

The standard surveillance practice in Akdeniz University Hospital was to follow up patients, who achieved complete remission or no evidence of disease following initial treatment, every three months for two years, every six months for the next three years, and then annually. The patients will continue to be followed-up until the disease recurs or mortality occurs.

### Statistical analysis

The primary outcome was progression-free survival (PFS). The secondary outcomes were overall survival (OS) and time to progression (TTP). PFS was determined to be the time period between the date of surgery and the date of disease progression, or relapse or mortality from any cause. TTP was calculated as the time period between surgery and disease progression/recurrence, or fatality caused by EC or complications associated with the surgery. OS was determined by the time period between the date of surgery and the date of mortality from any cause. The surviving patients that were not exhibiting progression or relapse were censored at the date they were last known to be alive according to the PFS and TTP data. Patients that continued to live, regardless of whether they exhibited progression or relapse, were censored at the date they were last known to be alive according to the OS analysis (10). The log-rank test was used to compare the Kaplan-Meier curves for OS, TTP and PFS. The Cox proportional hazards model was used to obtain the hazard ratio, for the treatment comparison, and the 95% confidence interval (CI), unadjusted or adjusted, for all factors. The data are expressed as the median and range for continuous variables. Binary variables are presented as counts and percentages. When appropriate, groups were compared with either a Mann-Whitney U test or a χ^2^ test. All P-values were two-sided and P<0.05 was considered to indicate a statistically significant difference. For statistical analysis, the Stata software package (Special Edition v11.2 for Macintosh OSX; StataCorp, College Station, TX, USA) was used.

## Results

### Patient characteristics

**Figure 1:**
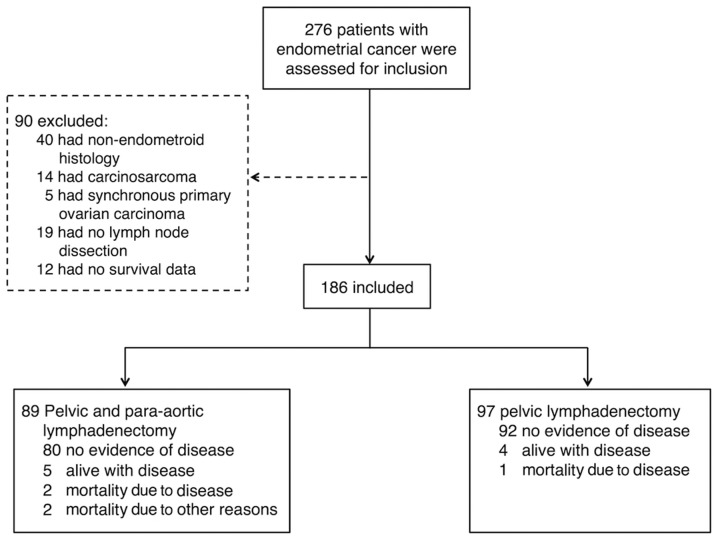
Study design.

A total of 276 patients were assessed for inclusion in the current study. Of these patients, 90 were excluded from the analysis: 40 had a non-endometrioid histology, 14 presented with carcinosarcoma, five had synchronous ovarian carcinoma, 19 had not undergone LN dissection and 12 had no survival data. Thus, analysis of a total of 186 patients, comprised of 97 patients in the PLND group and 89 in the PPaLND group (Fig. 1), was conducted. Table I compares the clinical and pathological characteristics of the lymphadenectomy groups. The PPaLND group was significantly older (median age, 59 vs. 55 years; P=0.0034), exhibited significantly larger tumor sizes (3.5 vs. 2.8 cm; P=0.0023), less laparoscopic surgery (6.7 vs. 32.0%; P<0.0001), more pelvic LNs removed (26 vs. 22; P=0.018), a greater number of para-aortic LNs removed (14 vs. 0; P<0.0001), an increased number of patients with stage II or more advanced disease (42.8% vs. 4.1%; P<0.0001), a greater number of HR patients (25.8 vs. 4.1%; P<0.0001) and more patients who received adjuvant treatment (75.6 vs. 34.4%; P<0.0001). The median follow-up time for all patients was 38 months (95% CI, 36.47–42.90).

Logistic regression analysis was performed to determine whether the differences between the groups influenced PFS (Table II). The risk of recurrence stratification was the only independent factor of PFS. Therefore, the lymphadenectomy groups were stratified according to GOG risk of recurrence stratification. Since there were few HR patients in the PLND group, the HIR and HR groups (HIR/HR), and the LIR and LR groups (LIR/LR) were combined.

Of the 119 LIR/LR patients, 78 underwent PLND and 41 underwent PPaLND. The PPaLND group included significantly more patients with stage II disease (24.4 vs. 0%; P=0.0167), fewer patients receiving laparoscopic surgery (4.9 vs. 33.3%; P<0.0001), a greater number of para-aortic LNs removed (15 vs. 0; P<0.0001) and more patients receiving adjuvant treatment when compared with the PLND group (55.0 vs. 20.5%; P<0.0001; Table III). Multivariate logistic regression analysis revealed no significant association between these covariates and PFS (Table IV). Of the 67 HIR/HR patients, 19 underwent PLND and 48 underwent PPaLND. The lymphadenectomy groups were comparable with regard to their baseline characteristics (Table V).

### Survival analysis

**Figure 2:**
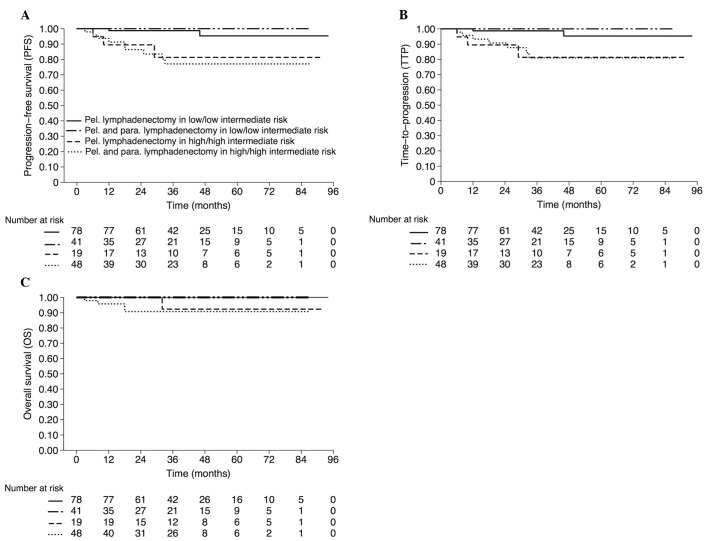
Estimated (A) progression-free survival rate, (B) time to progression and (C) overall survival rate stratified by lymphadenectomy status and risk of recurrence.

**Figure 3:**
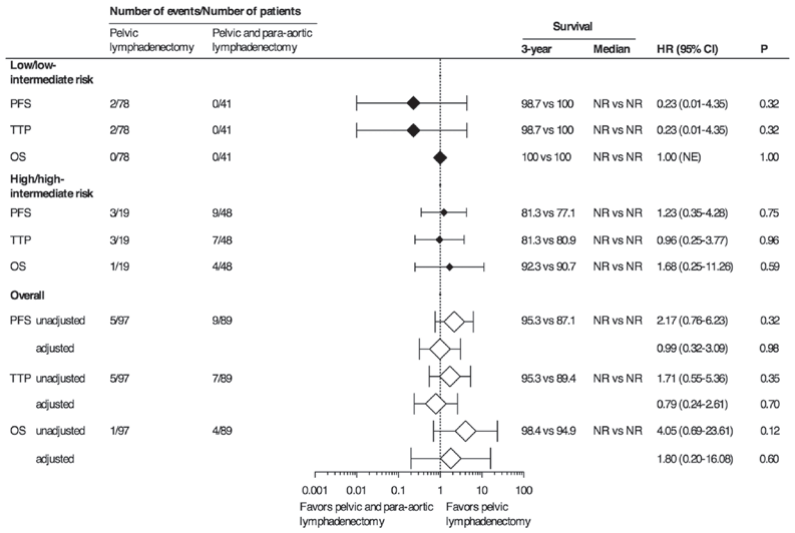
OS analyses (unadjusted and adjusted). HR, hazard ratio; CI, confidence interval; P, P-value; PFS, progression-free survival; TTP, time to progression; OS, overall survival; NR, not reached.

The estimated three-year OS, PFS and TTP rates for patients with LIR/LR stratified by lymphadenectomy groups were as follows: PLND, 100, 98.7 and 98.7%, respectively; and PPaLND, all 100%. The estimated three-year OS, PFS and TTP rates for patients with HIR/HR were as follows: PLND, 92.3, 81.3 and 81.3%; and PPaLND, 90.7, 77.1 and 80.9%, respectively (Fig. 2). No statistically significant differences were identified between three-year OS, PFS and TTP rates, regardless of the risk of recurrence stratification, between the lymphadenectomy groups (98.4, 95.3 and 95.3%, respectively for PLND; and 94.9, 87.1% and 89.4, respectively for PPaLND; Fig. 3).

## Discussion

A survival comparison of PLND and PPaLND was conducted in the present study in patients with endometrioid type EC according to the GOG risk of recurrence stratification. The results revealed no evidence of a survival advantage for PPaLND when compared with PLND in either of the LIR/LR or the HIR/HR patients. The aim of the present study was to histologically examine the outcomes in patients exhibiting endometrioid type EC. Histology is important, as the non-endometrioid EC subtypes have different immunophenotypes, natural histories and outcomes, which are determined by the tumor cell type (11,12).

Various studies have assessed the survival effect of lymphadenectomy in EC. The majority of studies have compared outcomes in patients who received a lymphadenectomy and those who did not. Trimble *et al* (13) noted a survival benefit among patients with stage I and grade 3 disease, but not those with grade 1 or grade 2 disease. Cragun *et al* (14) reported that patients who had >11 pelvic LNs removed exhibited significantly improved OS. However, aortic lymphadenectomy was not found to be beneficial in terms of improved survival in patients with apparent early-stage EC. In a large population-based analysis involving 42,184 patients, Smith *et al* (15) reported that lymphadenectomy conferred a disease-specific survival advantage. The improved disease-specific survival was most pronounced for patients with >11 LNs removed, or those with a disease stage II or higher.

However, the survival benefits observed in the above-mentioned studies, particularly in the patients presenting with high-grade tumors or those who had a greater number of LNs removed, frequently result from the misinterpretation of the disease stage. A certain proportion of patients who do not receive lymphadenectomy may be expected to develop LN metastases. Therefore, the outcomes of those studies reflect the difference in survival between the patients that are surgically identified to be true early-stage without LN involvement and those who are presumed to be early-stage with an unknown LN status.

Recently, the results of two large RCTs revealed that there was no significant survival benefit of a lymphadenectomy in patients with presumed early-stage EC (6,7). However, certain concerns regarding these trials have been raised, including the number of LNs removed and the inclusion of patients with LR of LN involvement. In particular, in the ASTEC trial (7), despite the lymphadenectomy group consisting of more patients with high-risk and advanced disease, radiotherapy was administered to an equal number of patients in each group. This factor resulted in the overtreatment of the patients in the no lymphadenectomy group.

Studies that examined patients who had the pelvic nodes removed as well as the nodes in the para-aortic region have indicated a survival benefit of lymphadenectomy, particularly in patients with node-positive disease (3–5). However, these studies had certain limitations, including small sample sizes, non-standardized adjuvant treatment strategies, heterogeneous tumor histology and uncertain inclusion criteria. One retrospective study (16) observed a significantly longer OS period in a PPaLND group as compared with a PLND group (hazard ratio, 0.53; 95% CI, 0.38–0.76; P=0.0005). Furthermore, this association was observed in the subgroup of patients with intermediate risk or HR (P=0.0009). However, OS was not associated with lymphadenectomy type in the LR patient subgroup. One major concern regarding the results of the study by Todo *et al* (16) was the lack of uniformity between the adjuvant treatment procedures for the intermediate and HR patients. In the PPaLND cohort, adjuvant treatment was limited to chemotherapy. In the PLND cohort, the patients received radiotherapy or chemotherapy depending on the preference of the patient and the discretion of the physician. Therefore, whether the improved survival, particularly in the HR patients, was associated with the para-aortic lymphadenectomy itself or with the therapeutic effect of chemotherapy on occult metastases is unclear. Conversely, another retrospective cohort that compared PLND with PPaLND in intermediate or HR patients indicated an improved disease-free survival rate in the patients who underwent PLND (80 vs. 62%; P=0.02) (17). However, the OS values were not significantly different between the two groups (P=0.93); in addition, the PLND group was more likely than the PPaLND group to have received multimodal adjuvant treatment.

The strengths of the present study include the analysis of a single histological type, the administration of adjuvant treatment as determined by the risk of recurrence analysis proposed by the GOG, the employment of relatively uniform surgical procedures/techniques and the adequacy of staging performed by subspecialized gynecological oncologists. The limitations include potential entry bias with case-selection, the lack of uniformity in the adjuvant treatment of HR-stage III patients, the relatively short median follow-up time and the small number of HR patients, particularly in the PLND group.

Recommendations regarding the use of adjuvant treatment in HIR/HR patients with any histological subtype or in patients with any stage of non-endometrioid histology vary widely. The lack of standardization is apparent in previous studies (3–5,16,17). The current GOG-258 trial compares the combination of chemotherapy and radiotherapy with chemotherapy alone (18). The PORTEC-3 trial compares the combination of chemotherapy and radiation with radiation alone (19). These trials may aid with determining the most appropriate adjuvant treatment modality for patients with optimally resected HR disease.

Following clarification of these issues, the therapeutic effect of lymphadenectomy may be assessed more thoroughly. However, concerns regarding bias and the use of adjuvant treatment in the PLND alone arm of the trial remain; for example, selection of the adjuvant treatment to be administered in patients with positive pelvic nodes but unknown para-aortic node status. Prior studies have shown that over half of patients with positive pelvic nodes exhibit positive para-aortic LN metastases (20,21). Thus, the subjects would experience a 50% chance of concurrent para-aortic metastases or the other 50% may be overtreated. Notably, skip metastases, the occurence of isolated para-aortic LN metastases in individuals with negative pelvic nodes, has been reported in ~1% surgically staged EC patients (22,23). In the current study, this rate was 3.3%. Determining which adjuvant therapy is administered to patients with negative pelvic nodes and whether the 1–3% likelihood of skip metastases is negligible requires further analysis.

In conclusion, investigating the therapeutic effect of lymphadenectomy, particularly in HR patients, is not considered to be possible based on the findings of the present study. Although performing an extended lymphadenectomy may provide valuable prognostic data, the procedure is solely acceptable as an experimental determinant of therapeutic intent.

## Figures and Tables

**Table I:** Clinical and pathological characteristics of patients exhibiting endometrioid type endometrial cancer.

	Lymphadenectomy		
		
Variable	Pelvic, n=97	Pelvic and para-aortic, n=89	P-value
Median age at surgery, years (IQR)	55 (49–61)	59 (53–65)	0.003
Histologic subtype, n (%)			
Endometrioid, pure	67 (69.1)	45 (50.6)	0.023^a^
Endometrioid, with squamous differentiation	28 (28.9)	44 (49.4)	
Endometrioid, villoglandular variant	1 (1.0)	0 (0.0)	
Endometrioid, ciliated cell variant	1 (1.0)	0 (0.0)	
FIGO stage, n (%)			
IA	70 (72.2)	28 (31.5)	<0.001^b^
IB	23 (23.7)	23 (25.8)	
II	0 (0.0)	14 (15.7)	
IIIA	0 (0.0)	3 (3.4)	
IIIC	4 (4.1)	19 (21.3)	
IV	0 (0.0)	2 (2.2)	
Risk of recurrence, n (%)			
Low	57 (58.8)	21 (23.6)	<0.001^a^
Low-intermediate	21 (21.6)	20 (22.5)	
High-intermediate	15 (15.5)	25 (28.1)	
High	4 (4.1)	23 (25.8)	
Median tumor size, cm (IQR)	2.8 (1.5–4.0)	3.5 (2.3–5.0)	0.002
Peritoneal cytology positive, n (%)	2 (2.1)	3 (3.4)	0.670
Surgery, n (%)			
Laparoscopy	31 (32.0)	6 (6.7)	<0.001
Laparotomy	66 (68.0)	83 (93.3)	
Median lymph nodes removed, n (IQR)			
Pelvic lymph nodes	22 (18–29)	26 (21–32)	0.018
Para-aortic lymph nodes	0 (0.0)	14 (9–19)	<0.001
Adjuvant treatment, n (%)			
None	63 (64.9)	20 (22.5)	<0.001^a^
Radiotherapy alone	31 (32.0)	45 (50.6)	
Chemotherapy alone	0 (0.0)	2 (2.1)	
Chemotherapy and radiotherapy	2 (2.1)	15 (16.8)	
Unknown	1 (1.0)	7 (7.9)	
Median follow-up time, months (95% CI)	39 (38.14–47.13)	37 (31.88–41.06)	0.079

IQR, interquartile range; FIGO, International Federation of Gynecology and Obstetrics; CI, confidence interval. P-values following the Bonferroni correction were ^a^0.0125 and ^b^0.0083.

**Table II:** Univariate and multivariate logistic regression analysis of factors predicting progression or mortality in patients exhibiting endometrioid type endometrial cancer.

	Unadjusted			Adjusted		
		
Variable	HR	95% CI	P-value	HR	95% CI	P-value
Age at surgery, years						
<62	1			1		
≥62	5.55	1.75–17.63	0.004	1.57	0.47–5.26	0.460
Risk of recurrence						
Low/Low-intermediate	1			1		
High/High-intermediate	10.26	3.38–31.12	<0.001	9.42	1.21–73.62	0.032
Adjuvant therapy						
No	1			1		
Yes	3.74	1.20–11.61	0.023	0.96	0.13–6.97	0.970
Histologic subtype						
Endometrioid, pure	1					
Endometrioid, other	1.67	0.57–4.94	0.350	-	-	-
Tumor size, cm						
<4	1					
≥4	2.05	0.68–6.22	0.200	-	-	-
Peritoneal cytology						
Negative	1					
Positive	3.32	0.43–25.92	0.220	-	-	-
Surgery						
Laparoscopy	1					
Laparotomy	2.50	0.72–8.66	0.150	-	-	-
Lymph node dissection						
Pelvic lymph node dissection	1					
Pelvic and para-aortic lymph node dissection	2.21	0.73–6.73	0.150	-	-	-
Pelvic lymph nodes removed, n						
<25	1					
≥25	1.36	0.47–3.87	0.570	-	-	-

HR, hazard ratio; CI, confidence interval. Unadjusted and adjusted data, if any, were obtained by univariate and multivariate analyses, respectively.

**Table III:** Clinical and pathological characteristics of the patients with low/low-intermediate risk of endometrioid type endometrial cancer.

	Lymphadenectomy		
		
Variable	Pelvic, n=78	Pelvic and para-aortic, n=41	P-value
Median age at surgery, years (IQR)	54 (48–59)	50 (50–62)	0.061
Histologic subtype, N (%)			
Endometrioid, pure	57 (73.1)	25 (61.0)	0.180^a^
Endometrioid, with squamous differentiation	19 (24.3)	16 (39.0)	
Endometrioid, villoglandular variant	1 (1.3)	0 (0.0)	
Endometrioid, ciliated cell variant	1 (1.3)	0 (0.0)	
FIGO stage, n (%)			
IA	67 (85.9)	26 (63.4)	<0.001^b^
IB	11 (14.1)	5 (12.2)	
II	0 (0.0)	10 (24.4)	
Grade, n (%)			
I	69 (88.5)	34 (82.9)	0.330^b^
II	9 (11.5)	6 (14.6)	
III	0 (0.0)	1 (2.5)	
Myometrial invasion, n (%)			
<1/2	67 (85.9)	31 (75.6)	0.160
≥1/2	11 (14.1)	10 (24.4)	
Lymphovascular invasion, n (%)	2 (2.6)	1 (2.4)	0.970
Median tumor size, cm (IQR)	2.5 (1.0–3.5)	3.0 (2.0–4.0)	0.054
Peritoneal cytology positive, n (%)	0 (0.0)	1 (2.4)	0.350
Surgery, n (%)			
Laparoscopy	26 (33.3)	2 (4.9)	<0.001
Laparotomy	52 (66.7)	39 (95.1)	
Median lymph nodes removed, n (IQR)			
Pelvic lymph nodes	22 (18–27)	26 (21–31)	0.080
Para-aortic lymph nodes	0 (0.0)	15 (9–19)	<0.001
Adjuvant treatment, n (%)			
None	62 (79.5)	18 (43.9)	<0.001^a^
Radiotherapy alone	16 (20.5)	21 (51.1)	
Chemotherapy alone	0 (0.0)	0 (0.0)	
Chemotherapy and radiotherapy	0 (0.0)	1 (2.5)	
Unknown	0 (0.0)	1 (2.5)	
Median follow up time, months (95% CI)	38 (36.91–46.81)	36 (31.37–46.14)	0.440

IQR, interquartile range; FIGO, International Federation of Gynaecology and Obstetrics; CI, confidence interval. P-values following the Bonferroni correction were ^a^0.0125 and ^b^0.0167.

**Table IV:** Univariate and multivariate logistic regression analysis of factors predicting progression or fatality in patients with low/low-intermediate risk endometrioid type endometrial cancer.

	Unadjusted			Adjusted		
		
Variable	HR	95% CI	P-value	HR	95% CI	P-value
Adjuvant therapy						
No	1					
Yes	2.87	0.13–62.0	0.500	-	-	-
Surgery						
Laparoscopy	1					
Laparotomy	3.88	0.17–90.93	0.400	-	-	-
FIGO stage						
I	1					
II	0.33	0.01–39.95	0.650	-	-	-
Age at surgery, years						
<54	1					
≥54	5.58	0.33–93.37	0.230	-	-	-
Tumor size, cm						
<2.5	1					
≥2.5	0.18	0.01–3.01	0.240	-	-	-
Pelvic lymph nodes removed						
<25	1					
≥25	1.20	0.07–19.39	0.900	-	-	-

HR, hazard ratio; CI, confidence interval; FIGO, International Federation of Gynecology and Obstetrics. Unadjusted and adjusted data, if any, were obtained by univariate and multivariate analyses, respectively.

**Table V:** Clinical and pathological characteristics of the patients with high/high-intermediate risk endometrioid histological subtype of endometrial cancer.

	Lymphadenectomy		
		
Variable	Pelvic, n=19	Pelvic and para-aortic, n=48	P-value
Median age at surgery, years (IQR)	62 (52–70)	62 (54–67)	0.600
Histologic subtype, n (%)			
Endometrioid, pure	10 (52.6)	20 (41.7)	0.420^a^
Endometrioid, with squamous differentiation	9 (47.4)	28 (58.3)	
Endometrioid, villoglandular variant	0 (0.0)	0 (0.0)	
Endometrioid, ciliated cell variant	0 (0.0)	0 (0.0)	
FIGO stage, n (%)			
IA	3 (15.8)	2 (4.2)	0.100^b^
IB	12 (63.2)	18 (37.5)	
II	0 (0.0)	4 (8.3)	
IIIA	0 (0.0)	3 (6.2)	
IIIC	4 (21.0)	19 (39.6)	
IVB	0 (0.0)	2 (4.2)	
Grade, n (%)			
I	3 (15.8)	9 (18.8)	0.170^c^
II	14 (73.7)	24 (50.0)	
III	2 (10.5)	15 (31.2)	
Myometrial invasion, n (%)			
<1/2	4 (21.0)	6 (12.5)	0.450
≥1/2	15 (78.9)	42 (87.5)	
Lymphovascular invasion, n (%)	6 (31.6)	19 (39.6)	0.540
Median tumor size, cm (IQR)	3.5 (3.0–4.0)	3.5 (2.5–5.3)	0.600
Peritoneal cytology positive, n (%)	2 (10.5)	2 (4.2)	0.320
Surgery, n (%)			
Laparoscopy	5 (26.3)	4 (8.3)	0.110
Laparotomy	14 (73.7)	44 (91.7)	
Median lymph nodes removed, n (IQR)			
Pelvic lymph nodes	27 (17–32)	26 (22–32)	0.600
Paraaortic lymph nodes	0 (0.0)	13 (8–20)	<0.001
Adjuvant treatment, n (%)			
None	1 (5.3)	2 (4.2)	0.210^a^
Radiotherapy alone	15 (78.9)	24 (50.0)	
Chemotherapy alone	0 (0.0)	2 (4.2)	
Chemotherapy and radiotherapy	2 (10.5)	14 (29.1)	
Unknown	1 (5.3)	6 (12.5)	
Median follow-up time, months (95% CI)	39 (34.29–57.40)	39 (28.60–40.44)	0.140

IQR, interquartile range; FIGO, International Federation of Gynaecology and Obstetrics; CI, confidence interval. P-values following the Bonferroni correction were ^a^0.0125, ^b^0.0083 and ^c^0.0167.
